# Supplementary material for: SAMURAI: shallow analysis of copy number alterations using a reproducible and integrated bioinformatics pipeline
Source: Brief Bioinform. 2025 Jan 29;26(1):bbaf035. doi: 10.1093/bib/bbaf035 (PMC11775468; doi:10.1093/bib/bbaf035)
Supplement: SAMURAI_SUPPLEMENTARY_FINAL_bbaf035 [file samurai_supplementary_final_bbaf035.docx]

Supplementary Results

SAMURAI: Shallow Analysis of copy nuMber alterations Using a Reproducible And Integrated bioinformatics pipeline

Sara Potente^1^, Diego Boscarino^2^, Dino Paladin^2^, Sergio Marchini^3^, Luca Beltrame^3,^*, Chiara Romualdi^1,^*

1 Department of Biology, University of Padova, Italy

2 AB Analitica S.r.l., Padova, Italy

3 IRCCS Humanitas Research Hospital, Rozzano, Milano, Italy.

*Co-last authors

**Correspondence to:**

Luca Beltrame

IRCCS Humanitas Research Hospital

E-mail: luca.beltrame@humanitasresearch.it

Phone: +39-02-8244-5243

| **Sample** | **Mean Depth** | **Median** **Depth** |
| --- | --- | --- |
| Sample A1 | 0.8919X | 1X |
| Sample B07 | 0.6246X | 0X |
| Sample C05 | 0.446X | 0X |
| Sample D03 | 0.2675X | 0X |
| Sample E01 | 0.0891X | 0X |

### **Supplementary Table S1.** Table reporting the different depths of *in-silico* diluted samples (see Materials and Methods). Sample A1 is the original sample without dilution.

| **Sample** | **Mean Depth** | **Median Depth** |
| --- | --- | --- |
| SM-74_01 | 0.3110X | 0X |
| SM-74_02 | 0.0548X | 0X |
| SM-74_03 | 0.1866X | 0X |
| SM-74_04 | 0.3110X | 0X |
| SM-74_05 | 0.0548X | 0X |
| SM-74_06 | 0.1866X | 0X |
| SM-74_07 | 0.3110X | 0X |
| SM-74_08 | 0.0548X | 0X |
| SM-74_09 | 0.1866X | 0X |
| SM-74_10 | 0.3110X | 0X |
| SM-74_11 | 0.0548X | 0X |
| SM-74_12 | 0.1866X | 0X |
| SM-74_13 | 0.3110X | 0X |
| SM-74_14 | 0.0548X | 0X |
| SM-74_15 | 0.1866X | 0X |
| SM-74_16 | 0.3110X | 0X |
| SM-74_17 | 0.0548X | 0X |
| SM-74_18 | 0.1866X | 0X |
| SM-74_19 | 0.3110X | 0X |
| SM-74_20 | 0.0548X | 0X |
| SM-74_21 | 0.1866X | 0X |
| SM-74_22 | 0.3110X | 0X |
| SM-74_23 | 0.0548X | 0X |
| SM-74_24 | 0.1866X | 0X |
| SM-74_25 | 0.3110X | 0X |
| SM-74_26 | 0.0548X | 0X |
| SM-74_27 | 0.1866X | 0X |
| SM-74_28 | 0.3110X | 0X |
| SM-74_29 | 0.0548X | 0X |
| SM-74_30 | 0.1866X | 0X |

### **Supplementary Table S2.** Mean and median coverage for *in-silico* dilutions of synthetic normal data (see Materials and Methods).


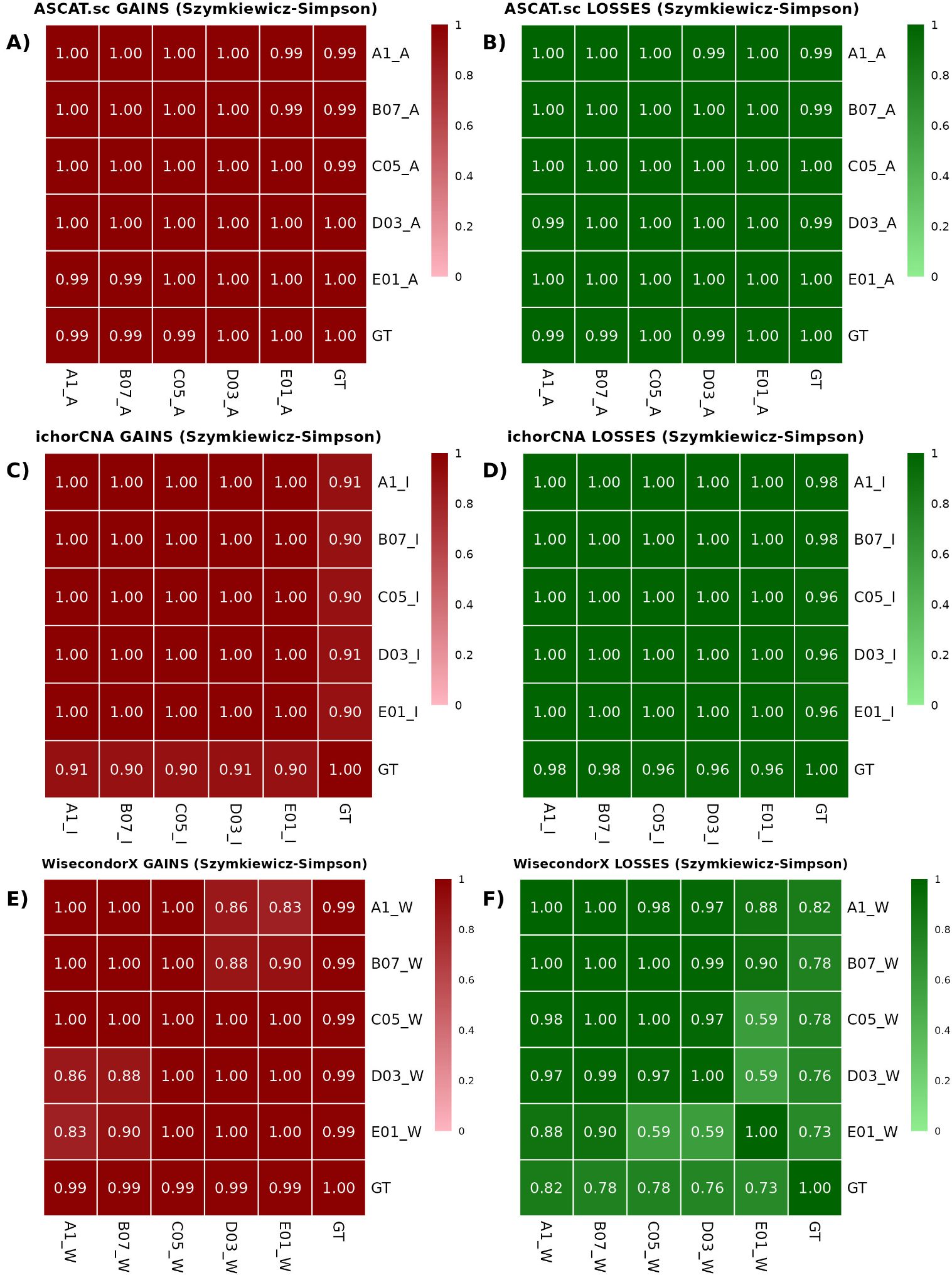


### **Supplementary Figure S1.** Heatmap reporting the Szymkiewicz-Simpson coefficient metrics for the comparison between inferred copy numbers and ground truth copy numbers in data set S. A) Metrics for gains inferred from ASCAT.sc versus ground truth. B) Metrics for losses inferred from ASCAT.sc versus ground truth. C) Metrics for gains inferred from ichorCNA versus ground truth. D) Metrics for losses inferred from ichorCNA versus ground truth. E) Metrics for gains inferred from Wisecondorx versus ground truth. F) Metrics for losses inferred from WisecondorX versus ground truth. Original sample: A1, *in-silico* diluted samples: B07, C05, D03, E01. Color intensity within the heatmap reflects the coefficient value, with higher values indicating better agreement.

| **HGSOC** | | |
| --- | --- | --- |
| **Sample ID** | **GAINS** | **LOSSES** |
| 1 | 0,97 | 0,99 |
| 10 | 0,92 | 1,00 |
| 113_left | 0,97 | 0,96 |
| 113_right | 0,99 | 0,95 |
| 119 | 0,88 | 0,97 |
| 12 | 0,99 | 1,00 |
| 123_left | 0,97 | 0,96 |
| 123_right | 0,93 | 0,73 |
| 131_left | 0,81 | 0,64 |
| 131_right | 0,91 | 0,97 |
| 133 | 0,97 | 0,99 |
| 137 | 1,00 | 0,96 |
| 148 | 1,00 | 0,98 |
| 164 | 0,98 | 0,98 |
| 183_left | 0,86 | 0,82 |
| 183_right | 0,97 | 0,47 |
| 186 | 0,99 | 0,08 |
| 202_left | 1,00 | 0,99 |
| 202_right | 0,98 | 0,99 |
| 203 | 0,99 | 0,98 |
| 206 | None (SAMURAI) | 0,00 |
| 207 | 0,96 | 0,62 |
| 21 | 0,97 | 0,99 |
| 212 | 1,00 | 0,99 |
| 219 | 0,99 | 0,91 |
| 31 | 0,91 | 0,69 |
| 31bis | 0,98 | 0,68 |
| 31tris | 0,93 | 0,98 |
| 4 | 0,98 | 0,99 |
| 41 | 0,91 | 0,99 |
| 45 | 0,98 | 0,00 |
| 5 | 0,95 | 0,95 |
| 50 | 1,00 | 0,96 |
| 51 | 0,98 | 0,06 |
| 8 | 0,97 | 1,00 |
| 89 | 0,92 | 1,00 |
| 99 | 1,00 | 0,99 |
| T15 | 1,00 | 0,99 |
| T20 | 0,97 | 0,99 |
| T25 | 0,90 | 0,79 |
| T37 | 0,98 | 1,00 |
| T41 | 0,94 | 0,99 |
| T5 | 0,99 | None (SAMURAI) |
| T59 | 0,16 | 0,36 |
| T6 | 0,95 | 0,98 |
| T62 | 0,99 | 0,76 |
| T9 | 0,82 | 0,98 |
| **LGSOC** | | |
| **Sample ID** | **GAINS** | **LOSSES** |
| 106 | 1,00 | 1,00 |
| 13 | 0,99 | 1,00 |
| 151 | 1,00 | 1,00 |
| 156 | 1,00 | None |
| 161_left | 0,00 | 0,00 |
| 161_right | 0,00 | 0,20 |
| 175 | 0,79 | 0,99 |
| 18 | None (SAMURAI) | 0,00 |
| 187_left | 1,00 | 0,98 |
| 187_right | 1,00 | 0,99 |
| 20 | 0,97 | 0,89 |
| 205 | 0,99 | 0,98 |
| 215 | 0,94 | None (Data set T) |
| 26 | None (SAMURAI) | None (Data set T) |
| 3 | 0,92 | 0,96 |
| 39 | 1,00 | 0,99 |
| 52 | 1,00 | 1,00 |
| 75 | 0,68 | 0,14 |
| T27 | 1,00 | 0,02 |
| T32 | 0,00 | 0,03 |
| **EC** | | |
| **Sample ID** | **GAINS** | **LOSSES** |
| 100 | 0,92 | 0,08 |
| 101 | 1,00 | 0,61 |
| 104 | 0,92 | 0,98 |
| 105 | None | None (Data set T) |
| 107 | 1,00 | 0,87 |
| 11 | 0,88 | 0,00 |
| 111 | 0,00 | 0,65 |
| 112 | 0,74 | 0,00 |
| 114 | 1,00 | 1,00 |
| 116 | 0,60 | 0,03 |
| 117 | 0,17 | 0,26 |
| 127 | 0,95 | 0,82 |
| 129 | None (Data set T) | 0,25 |
| 134 | 0,98 | None (Data set T) |
| 147 | 0,98 | 0,89 |
| 149 | 0,86 | 0,97 |
| 150 | 0,43 | 1,00 |
| 154 | 0,97 | 0,83 |
| 158 | 0,84 | 0,96 |
| 159 | 1,00 | 1,00 |
| 163 | 0,86 | 0,65 |
| 17 | None (Data set T) | 0,00 |
| 178 | 1,00 | 0,73 |
| 179 | 1,00 | 0,75 |
| 180 | 0,40 | 0,09 |
| 182 | 1,00 | 1,00 |
| 184 | 0,97 | 0,99 |
| 185 | 0,00 | 0,04 |
| 188 | 1,00 | 1,00 |
| 188bis | 0,94 | 0,98 |
| 190 | 0,84 | 0,76 |
| 191 | 0,00 | 0,00 |
| 192 | 1,00 | 0,97 |
| 195 | 1,00 | 0,90 |
| 198 | 1,00 | 1,00 |
| 200 | 0,95 | None (SAMURAI) |
| 210 | 0,94 | 0,98 |
| 22 | 0,96 | 0,08 |
| 221 | 0,92 | 0,94 |
| 223 | 0,95 | 0,99 |
| 225 | 1,00 | 1,00 |
| 28 | 0,00 | 0,47 |
| 37 | None (Data set T) | None (Data set T) |
| 38 | 1,00 | None (Data set T) |
| 42 | None (Data set T) | 0,97 |
| 44 | 0,00 | None |
| 47 | 1,00 | None (Data set T) |
| 49 | 0,89 | 0,93 |
| 54 | 0,96 | 0,99 |
| 59 | 0,00 | 0,63 |
| 60 | 0,85 | 0,05 |
| 65 | 1,00 | 1,00 |
| 72 | 0,99 | 0,79 |
| 73 | 0,99 | 1,00 |
| 80 | 0,99 | 0,99 |
| 84 | 1,00 | None (Data set T) |
| 85 | 1,00 | 0,18 |
| 90 | 0,99 | 0,94 |
| 91 | 0,98 | 0,98 |
| 92 | 1,00 | None |
| 96 | 0,73 | 0,65 |
| 97 | 0,99 | 0,78 |
| 97bis | 1,00 | 0,00 |
| 98 | 1,00 | 0,86 |
| T10 | 0,73 | 0,63 |
| T16 | 0,00 | 0,00 |
| T2 | None (SAMURAI) | 0,13 |
| T22 | 0,97 | 0,95 |
| T23 | 0,69 | 1,00 |
| T24 | 0,97 | 0,00 |
| T28 | None (SAMURAI) | 0,38 |
| T29 | 0,00 | 0,00 |
| T3 | 0,99 | 0,29 |
| T33 | 1,00 | 0,97 |
| T4 | 1,00 | 0,99 |
| T42 | 0,95 | 0,99 |
| T47 | 0,96 | 0,99 |
| T48 | 0,99 | 0,00 |
| T52 | 1,00 | 0,96 |
| T55 | 0,61 | 0,97 |
| T57 | 0,98 | 0,69 |
| T8 | 0,97 | 0,98 |
| **MOC** | | |
| **Sample ID** | **GAINS** | **LOSSES** |
| 102 | 0,61 | 0,08 |
| 103 | None (Data set T) | 0,95 |
| 118 | 1,00 | 1,00 |
| 122 | 0,70 | 0,98 |
| 124 | 0,91 | 0,98 |
| 135 | None | None (Data set T) |
| 14 | 0,73 | 0,88 |
| 141 | None (SAMURAI) | 0,17 |
| 15 | 0,93 | 0,72 |
| 155 | 1,00 | 0,99 |
| 166 | None (SAMURAI) | 0,99 |
| 168 | 0,99 | 0,98 |
| 171 | 0,64 | 0,91 |
| 172 | 0,99 | 1,00 |
| 176 | 0,93 | 0,00 |
| 189 | 0,00 | 0,99 |
| 197 | None (SAMURAI) | None (Data set T) |
| 204 | None (Data set T) | 0,00 |
| 214 | 0,84 | 0,98 |
| 217 | 1,00 | 0,00 |
| 25 | 0,00 | None (Data set T) |
| 34 | 0,96 | 0,90 |
| 36 | 0,98 | 1,00 |
| 48 | 1,00 | 1,00 |
| 53 | 1,00 | 1,00 |
| 57 | 0,82 | 0,25 |
| 63 | 0,94 | 0,95 |
| 67 | 1,00 | None (Data set T) |
| 69 | None (SAMURAI) | 0,99 |
| 7 | 0,95 | 0,00 |
| T14 | 0,87 | 1,00 |
| T26 | 0,32 | 0,82 |
| T31 | 0,98 | 0,93 |
| T38 | 0,98 | 0,92 |
| T39 | 0,75 | 1,00 |
| T44 | 1,00 | 0,62 |
| T53 | 0,99 | 0,91 |
| T60 | 0,00 | 0,53 |
| **OCCC** | | |
| **Sample ID** | **GAINS** | **LOSSES** |
| 115 | 0,71 | 0,12 |
| 115bis | 1,00 | None (Data set T) |
| 126 | 0,99 | 1,00 |
| 128 | 0,98 | 0,82 |
| 132 | 0,13 | 0,27 |
| 16 | 0,97 | 0,98 |
| 174 | 0,81 | 0,98 |
| 181 | 0,94 | 0,97 |
| 193 | 0,98 | 0,00 |
| 208 | 0,99 | 0,98 |
| 213 | 0,98 | 0,98 |
| 220 | 0,95 | 0,97 |
| 224 | 0,94 | 0,95 |
| 23_left | 0,65 | 0,49 |
| 23_right | 1,00 | 0,78 |
| 29 | 0,98 | 0,99 |
| 33 | 0,97 | 1,00 |
| 6 | 0,75 | 0,79 |
| 70 | 1,00 | 0,99 |
| 78 | 0,97 | 0,95 |
| 82 | 1,00 | 1,00 |
| 83 | None (SAMURAI) | 1,00 |
| 86 | 1,00 | 0,98 |
| 95 | 0,88 | 0,98 |
| T1 | 0,98 | 0,98 |
| T13 | 1,00 | 0,96 |
| T19 | 0,00 | 0,53 |
| T36 | 0,98 | 0,75 |
| T49 | 0,98 | 0,99 |
| T54 | 1,00 | 0,98 |
| T56 | 1,00 | 0,16 |

### **Supplementary Table S3.** Overlap coefficient between data in data set T from original work and SAMURAI results for each sample in the cohort, divided by histotype. None indicates that no copy number alterations were found in both the original data and the SAMURAI analysis and thus a coefficient was not calculated; None (SAMURAI) indicates that it was due to no CNAs found by SAMURAI, and None (Data set T) due to no CNAs present in the original data set.


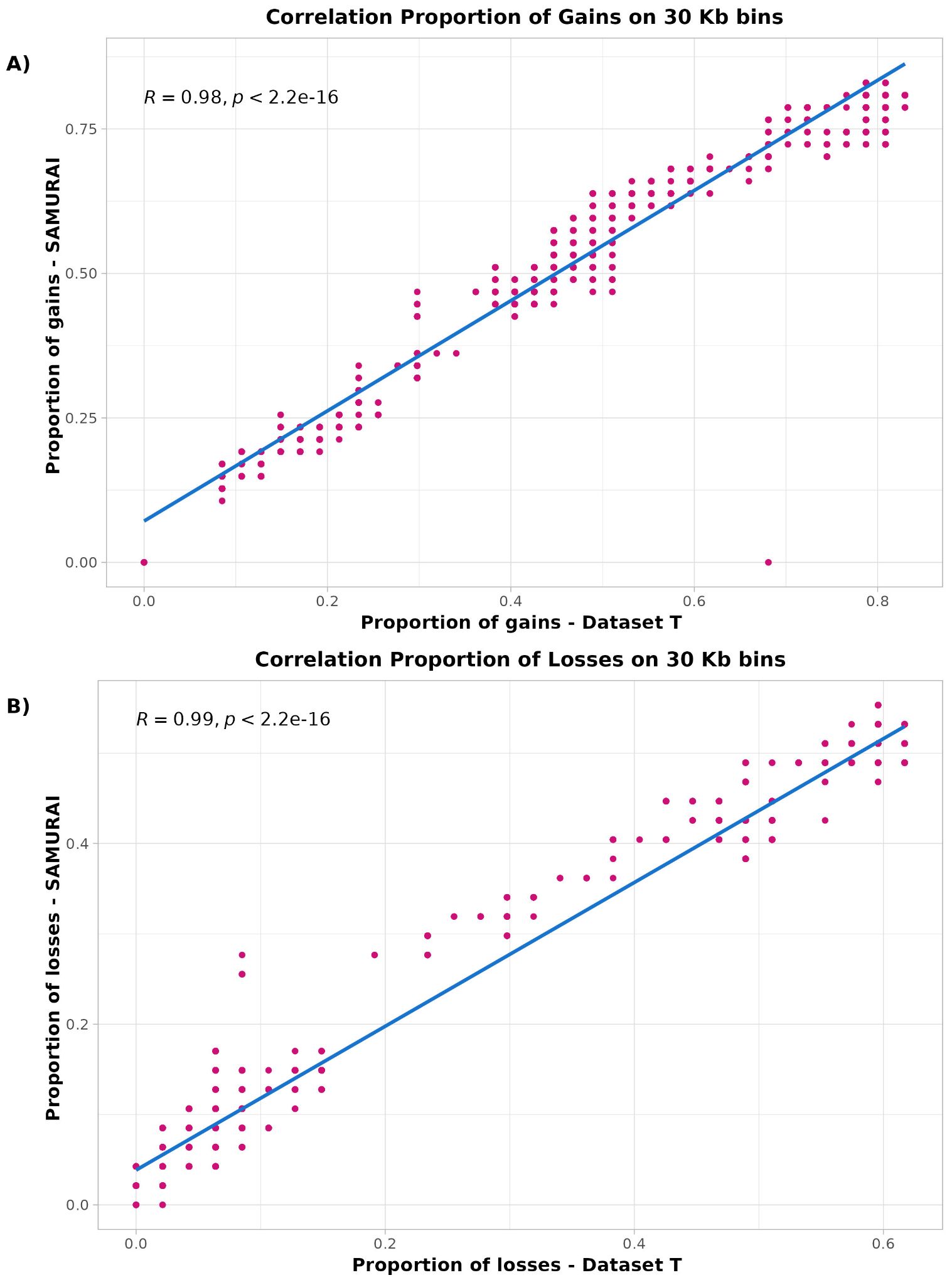


### **Supplementary Figure S2.** Scatterplot showing the correlation between proportion of gains and losses on overlapping 30 Kbp bins of chr8 between original segmentation files and SAMURAI’s segmentation files of HGSOC samples in data set T. **A)** Correlation between proportions of copy number gains. x axis, proportions from computed from original data; y axis, proportions from SAMURAI data. **B)** Correlation between proportions of copy number losses. x axis, proportions from computed from original data; y axis, proportions from SAMURAI data. R: Pearson Correlation coefficient; *p*: p-value.

###

###
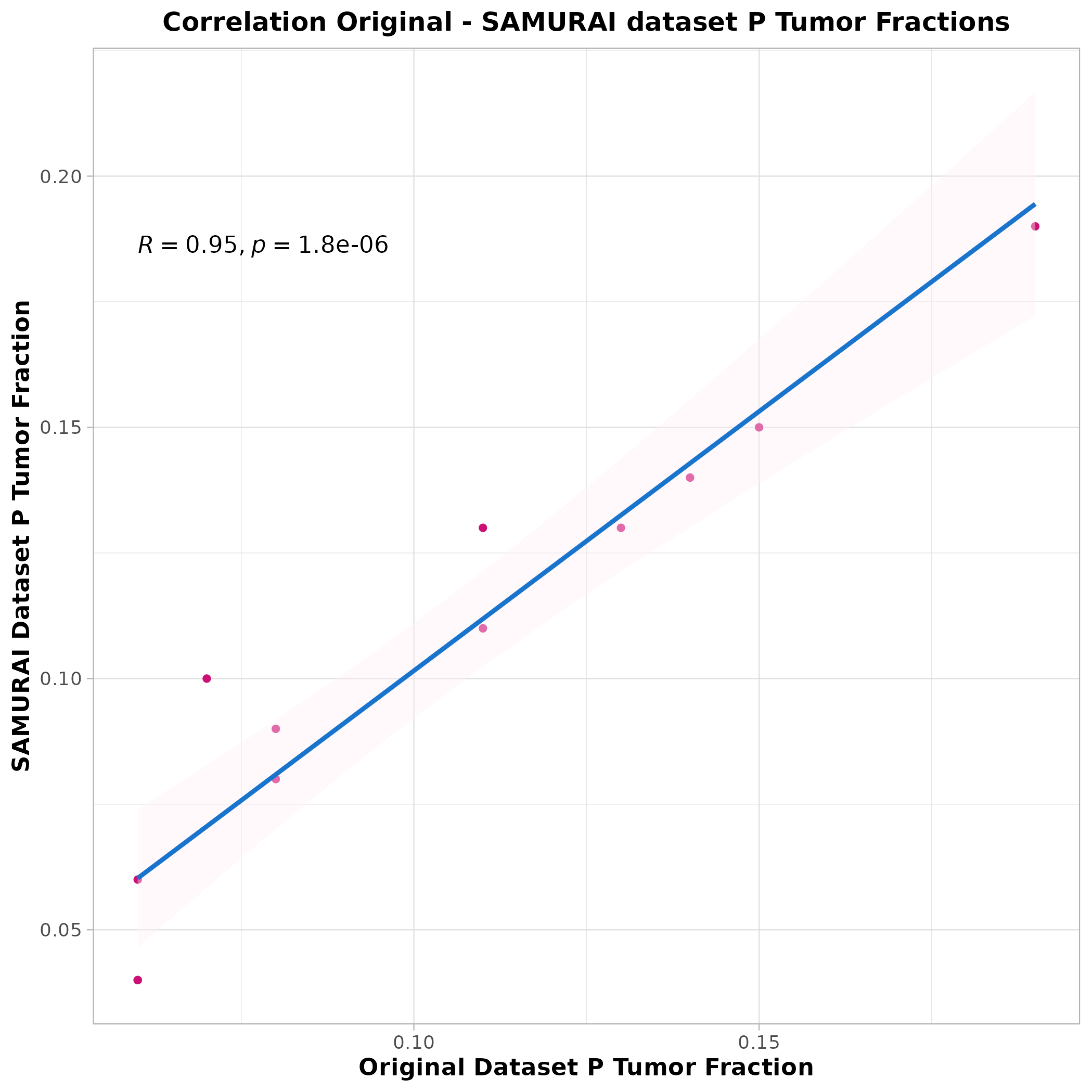


### **Supplementary Figure S3.** Scatterplot illustrating the correlation between tumor fractions (TFs) computed by SAMURAI with ichorCNA workflow and the original reported tumor fractions in data set P. X axis, original TFs from data set P; y axis, TFs computed by SAMURAI. R: Pearson Correlation coefficient; *p*: p-value.
