# Supplementary material for: SAMURAI: shallow analysis of copy number alterations using a reproducible and integrated bioinformatics pipeline
Source: Brief Bioinform. 2025 Jan 29;26(1):bbaf035. doi: 10.1093/bib/bbaf035 (PMC11775468; doi:10.1093/bib/bbaf035)
Supplement: SupplementaryFile2_ReportSAMURAI_Dataset_P_bbaf035 [file supplementaryfile2_reportsamurai_dataset_p_bbaf035.pdf]

multiqc

SAMURAI Results

SAMURAI's analysis results of Copy Number Alterations from shallow whole genome sequencing.

A modular tool to aggregate results from bioinformatics analyses across many samples into a single report.

> This report has been generated by the dincalcilab/samurai analysis pipeline.

Report generated on 2024-07-26, 15:24 CEST based on data in: `/home/incalci/lbeltrame/analysis/samurai-ichorcna/work/71/ce24b3ae34d027a022f66676b5e1a2`

General Statistics

Copy table | Configure columns | Scatter plot | Violin plot | Showing 12/12 rows and 2/6 columns. | Export as CSV

Sample Name	Insert Size	Mean Coverage
**21531-PL1_cfdna_ready**	147 bp	0.5 X
**21553-PL1_cfdna_ready**	173 bp	0.3 X
**21557-PL1_cfdna_ready**	164 bp	0.3 X
**21564-PL1_cfdna_ready**	146 bp	0.3 X
**21566-PL1_cfdna_ready**	139 bp	0.4 X

Sample Name	Insert Size	Mean Coverage
**21569-PL0_cfdna_ready**	162 bp	0.4 X
**21572-PL0_cfdna_ready**	159 bp	0.5 X
**21580-PL1_cfdna_ready**	141 bp	0.4 X
**21611-PL0_cfdna_ready**	167 bp	0.2 X
**21614-PL0_cfdna_ready**	165 bp	0.3 X
**21624-PL0_cfdna_ready**	168 bp	0.4 X
**21627-PL0_cfdna_ready**	168 bp	0.4 X

Picard

Picard is a set of Java command line tools for manipulating high-throughput sequencing data.

Alignment Summary

Please note that Picard's read counts are divided by two for paired-end data. Total bases (including unaligned) is not provided.

Percentages | Aligned Reads | Aligned Bases | Export Plot

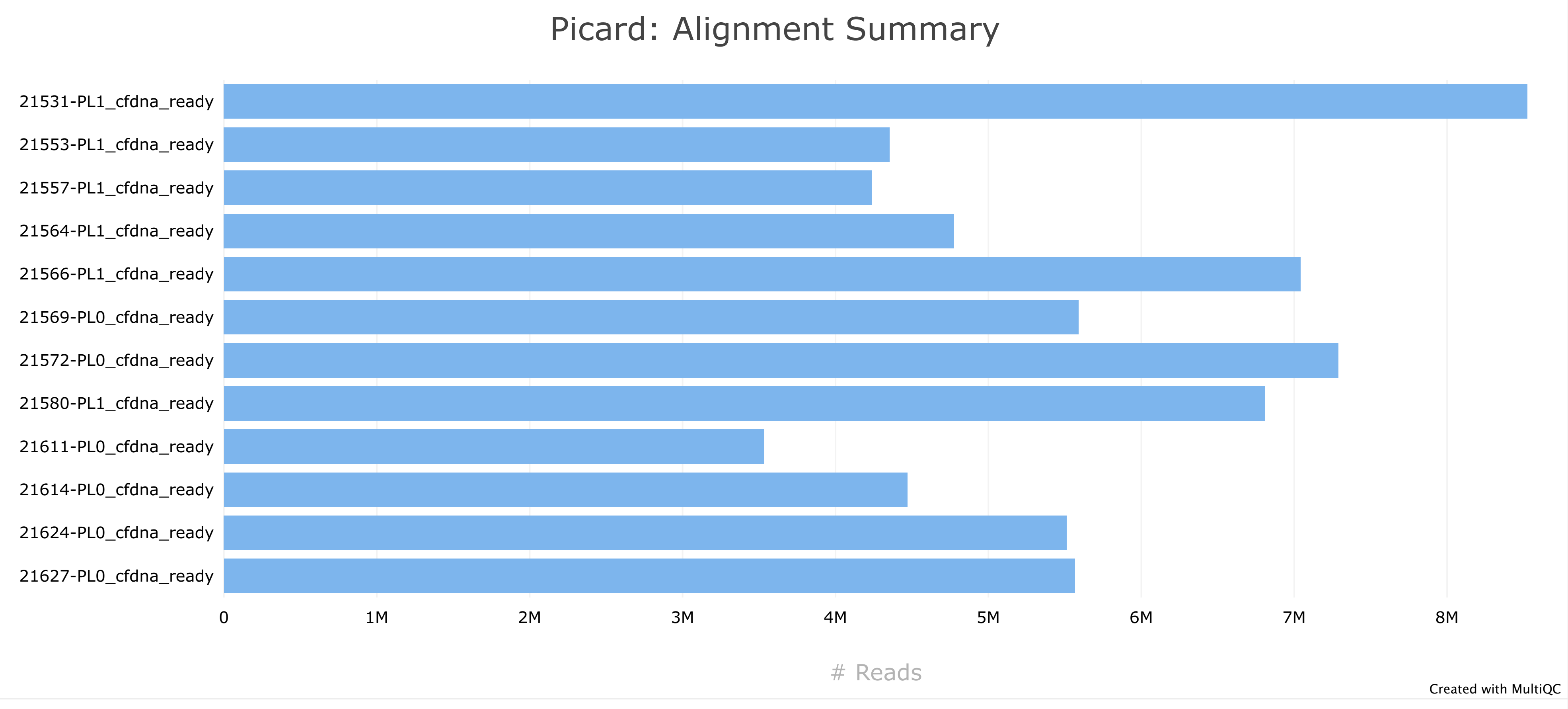

Mean read length

The mean read length of the set of reads examined.

Export Plot

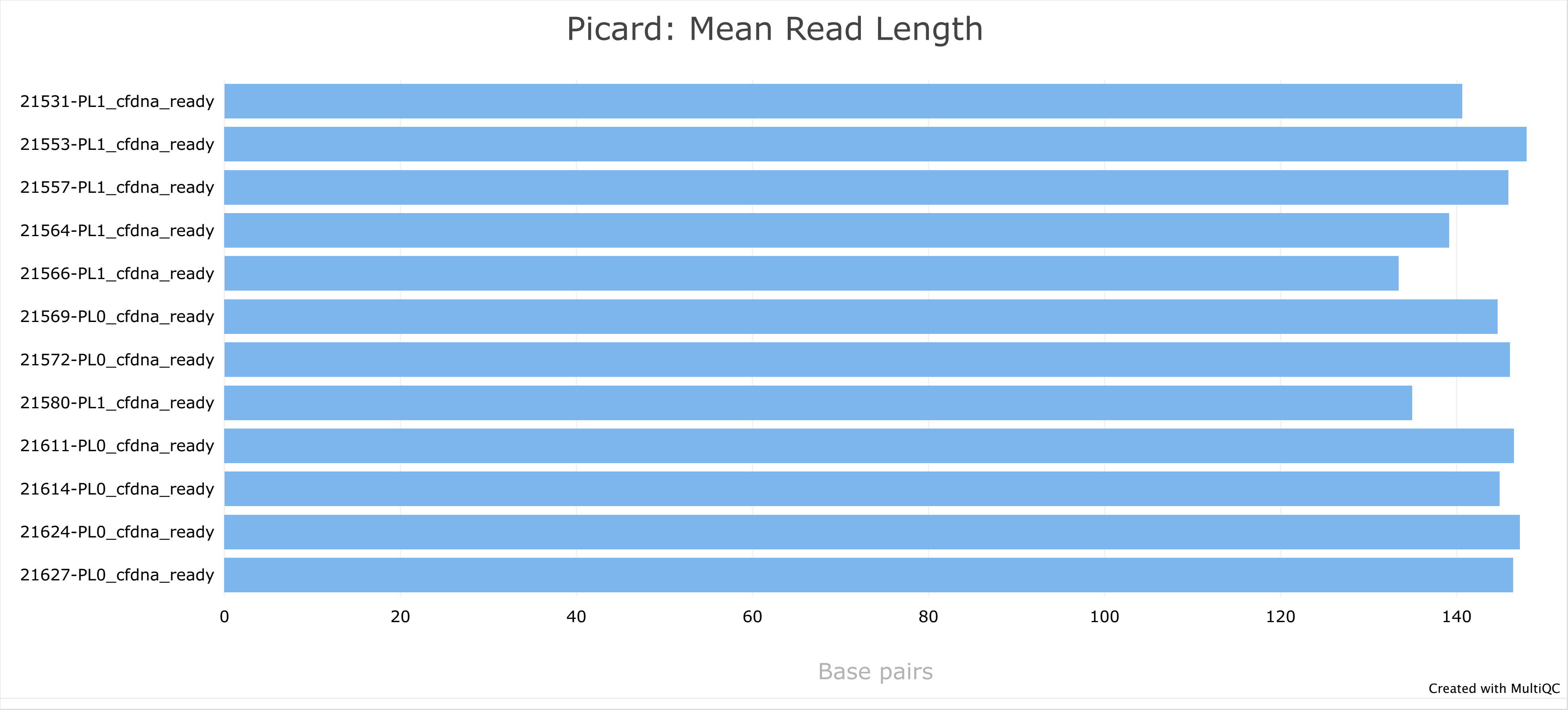

Base Distribution

Plot shows the distribution of bases by cycle.

% Adenine | % Cytosine | % Guanine | % Thymine | % Undetermined

Export Plot

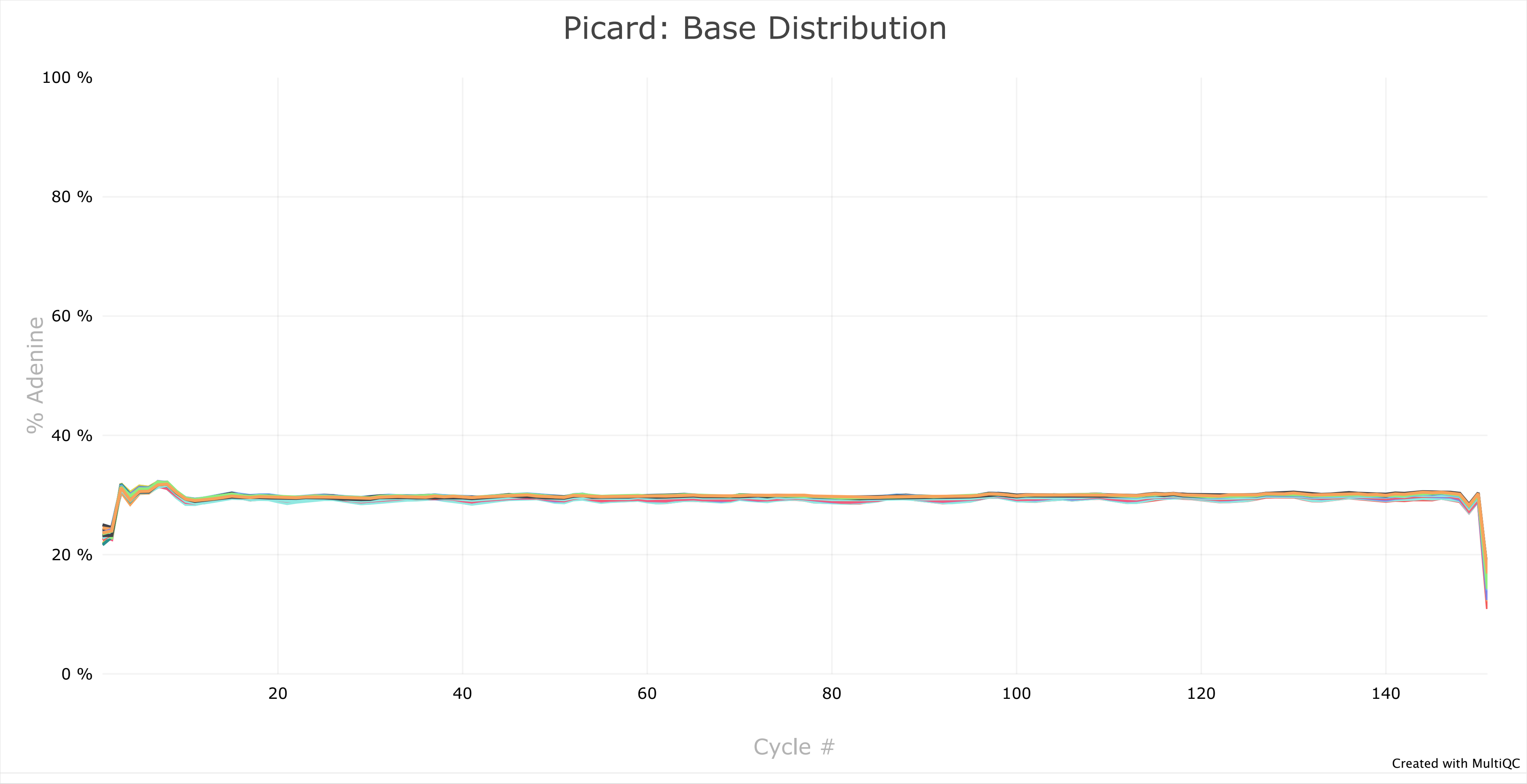

Insert Size

Plot shows the number of reads at a given insert size. Reads with different orientations are summed.

Counts | Percentages

Export Plot

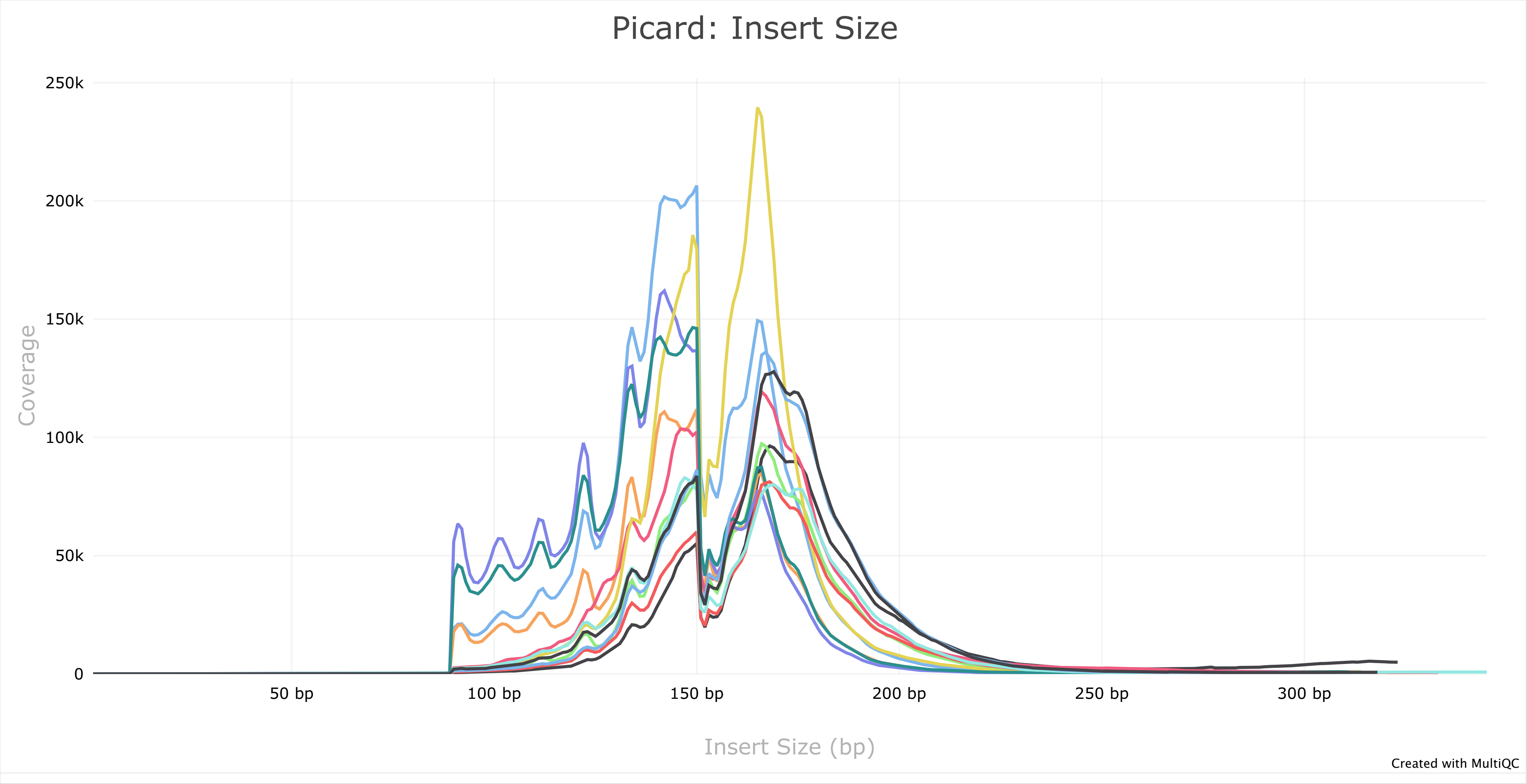

Mean Base Quality by Cycle

Plot shows the mean base quality by cycle.

Export Plot

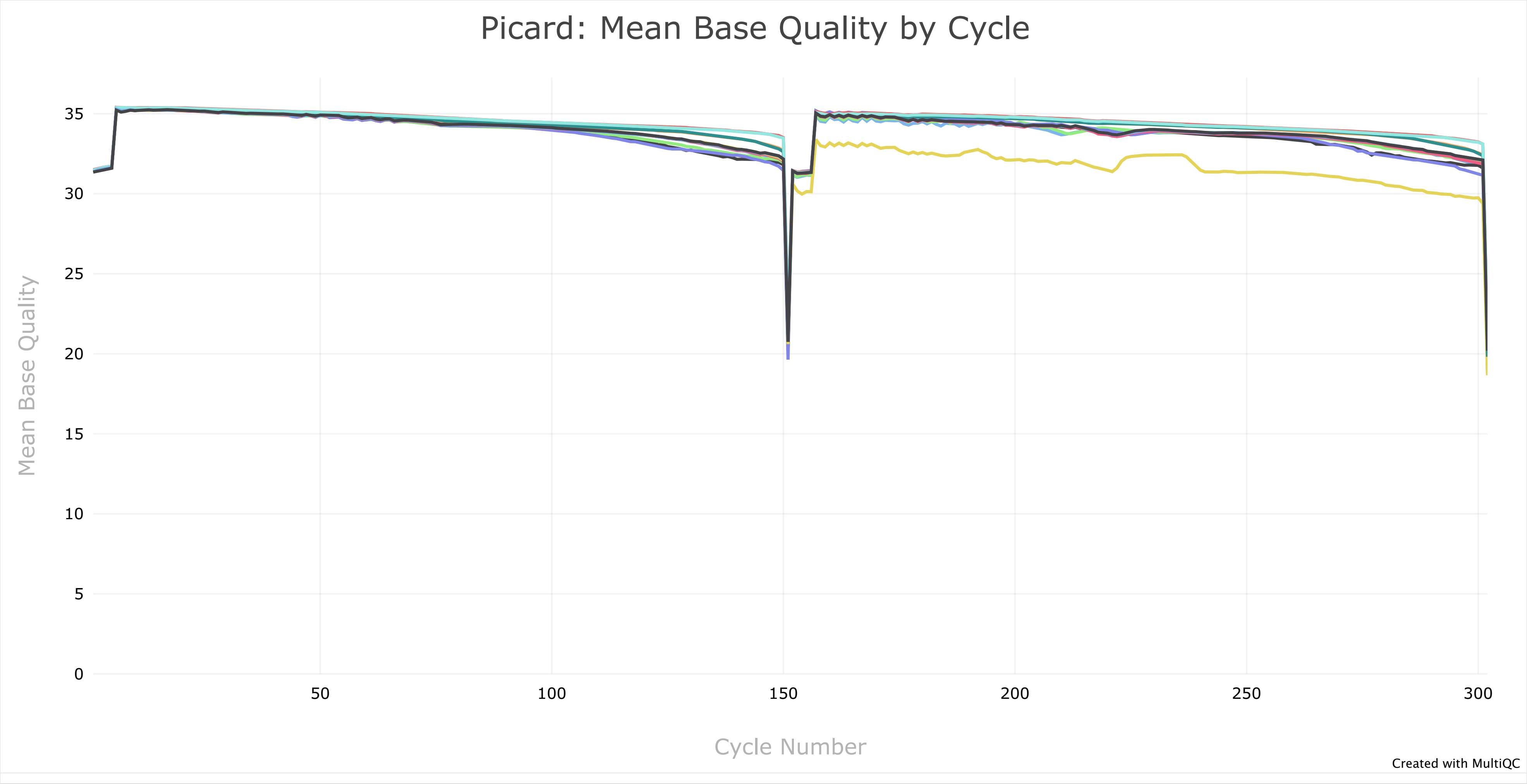

Base Quality Distribution

Plot shows the count of each base quality score.

Export Plot

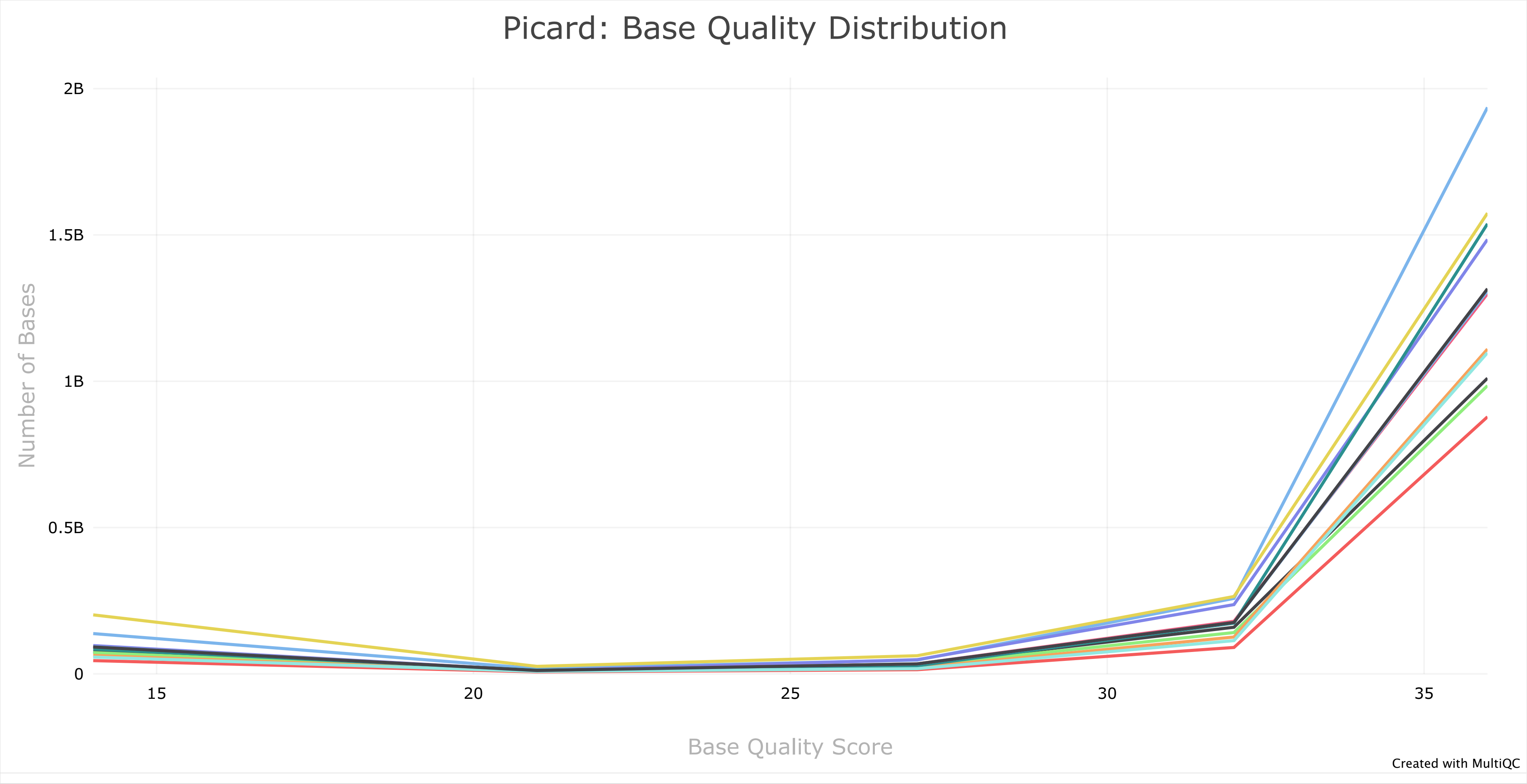

WGS Coverage

The number of bases in the genome territory for each fold coverage. Note that final 1% of data is hidden to prevent very long tails.

Percentage Drop-Off | Counts Histogram | Export Plot

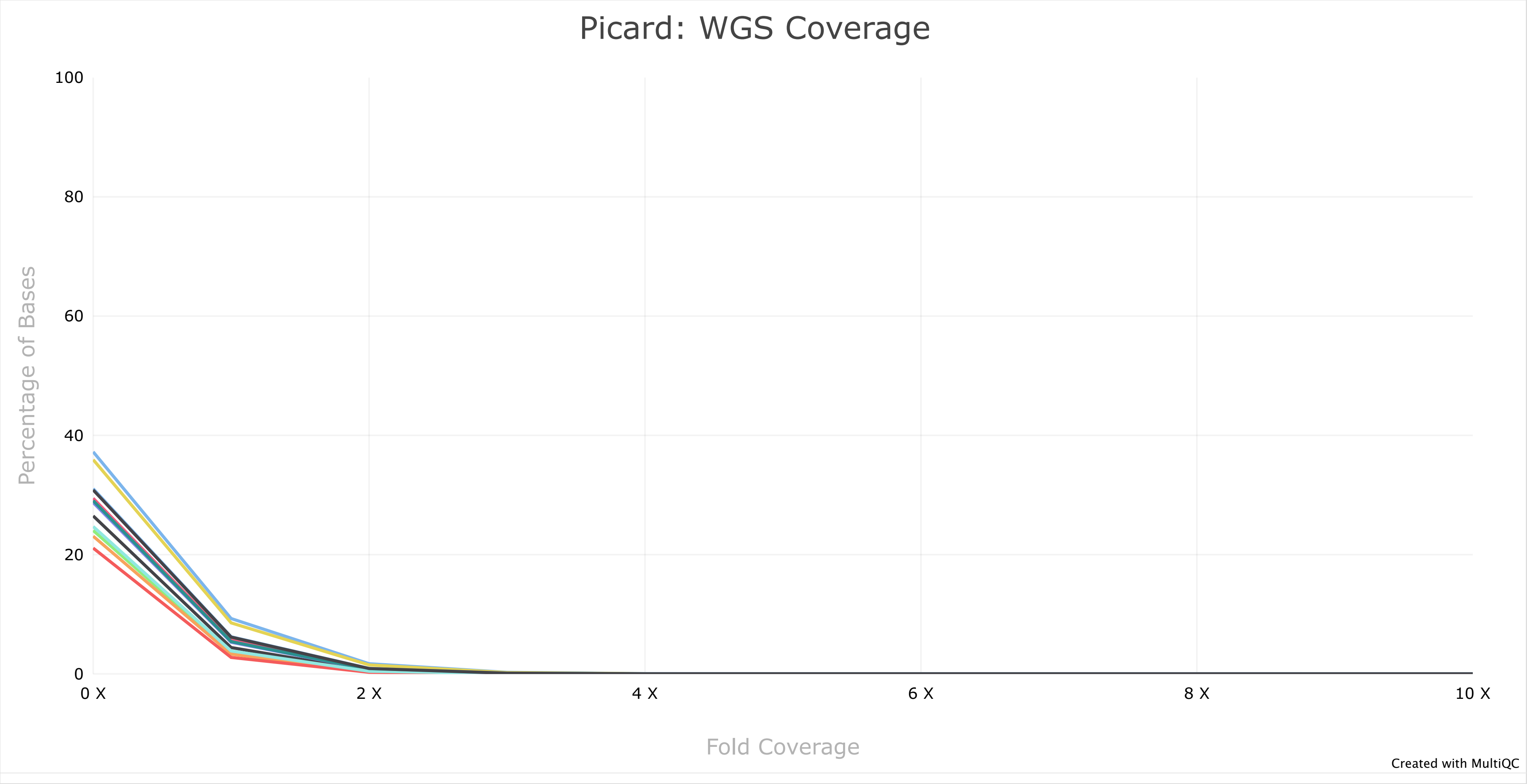

WGS Filtered Bases

For more information about the filtered categories, see the Picard documentation.

Export Plot

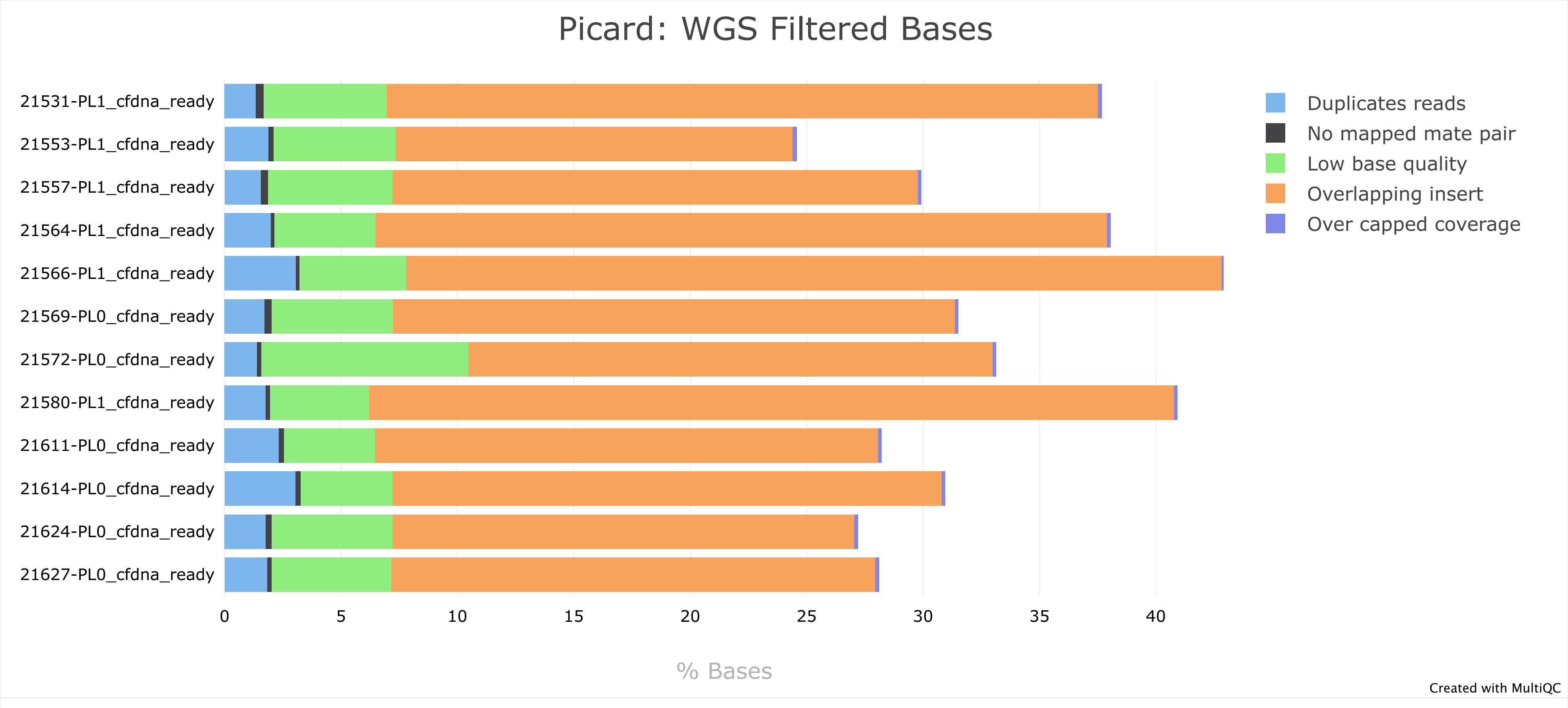

Gistic arm-level analysis summary

This table shows significant recurrent arm-level alterations identified by GISTIC2. Each alteration has an associated frequency and q-value for statistical significance. .

Copy table | Configure columns | Scatter plot | Violin plot | Showing 14/14 rows and 4/4 columns. | Export as CSV

Arm	Type of alteration	No. of samples	Frequency	q-value
**1p**	**Amp**	7	58.0	0.0002
**2p**	**Amp**	10	83.0	0.0000
**3q**	**Amp**	6	50.0	0.0028
**5p**	**Amp**	7	58.0	0.0002
**6p**	**Amp**	8	67.0	0.0000
**7q**	**Amp**	7	58.0	0.0002
**8q**	**Amp**	5	42.0	0.0222
**10p**	**Amp**	5	42.0	0.0108
**11q**	**Amp**	5	42.0	0.0249
**12p**	**Amp**	7	58.0	0.0002
**19p**	**Amp**	6	50.0	0.0028
**19q**	**Amp**	6	50.0	0.0032
**20p**	**Amp**	6	50.0	0.0009
**20q**	**Amp**	8	67.0	0.0000

IchorCNA

This table shows the tumor fraction and ploidy estimates made by ichorCNA.

Copy table | Configure columns | Scatter plot | Violin plot | Showing 12/12 rows and 3/3 columns. | Export as CSV

Sample ID	Ploidy	Tumor fraction	MAD
**21614-PL1**	2	0.04437	**0.0530**
**21553-PL1**	2	0.0419	**0.0513**
**21557-PL1**	2	0.08452	**0.0510**
**21627-PL1**	2	0.06006	**0.0478**
**21611-PL1**	2	0.1332	**0.0584**
**21572-PL1**	2	0.0922	**0.0461**
**21624-PL1**	2	0.1086	**0.0488**
**21566-PL1**	2	0.1358	**0.0540**
**21569-PL1**	2	0.1854	**0.0528**
**21531-PL1**	2	0.1291	**0.0445**
**21580-PL1**	2	0.1451	**0.0531**
**21564-PL1**	2	0.09609	**0.0557**

Gistic Analysis

Maftools genomic plot with segments highlighting significant Amplifications and Deletions.

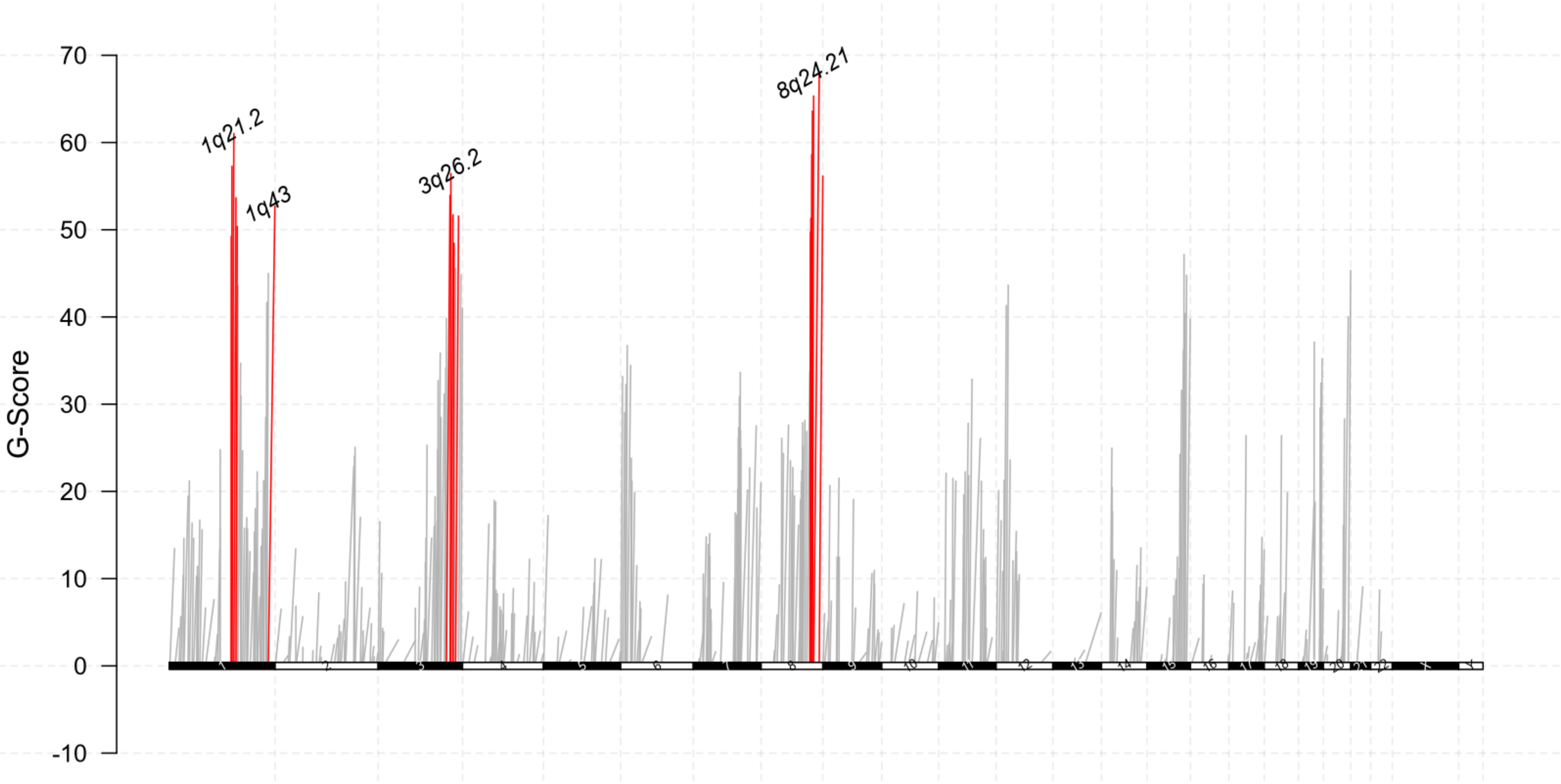

Gistic Analysis Summary

This table shows significant lesions identified by GISTIC2 analysis. Focal lesions are alterations which span less than 25% of a chromosomal arm. Broad lesions span more than 25% of the chromosomal arm, but less than a whole arm .

Copy table | Configure columns | Scatter plot | Violin plot | Showing 4/4 rows and 9/9 columns. | Export as CSV

Chromosome	Chromosome	Cytoband	Start	End	Length	CN Kind	Lesion Frequency	№ of Samples	Type of Lesion
**Amp_1q21.2_chr1:119990001-168510000**	chr1	1q21.2	119 990 001	168 510 000	48 519 999	**Amp**	1.00	11	focal
**Amp_1q43_chr1:218990001-248956422**	chr1	1q43	218 990 001	248 956 422	29 966 421	**Amp**	0.55	6	focal
**Amp_3q26.2_chr3:138490001-198295559**	chr3	3q26.2	138 490 001	198 295 559	59 805 558	**Amp**	1.00	11	broad
**Amp_8q24.21_chr8:107490001-145138636**	chr8	8q24.21	107 490 001	145 138 636	37 648 635	**Amp**	1.00	11	broad

Software Versions

Software Versions lists versions of software tools extracted from file contents.

Copy table

Group	Software	Version
AGGREGATE_ICHORCNA_TABLE	assemble_outputs	0.0.1
ASSEMBLE_GISTIC_OUTPUT	gistic-cli	0.4.2
CONCATENATE_BIN_PLOTS	qpdf	11.3.0
CORRECT_LOGR_ICHORCNA	correct_logR_ichorcna	0.1
GISTIC2	GISTIC2	0.1
HMMCOPY_READCOUNTER_ICHORCNA	hmmcopy	0.1.1
MAFTOOLS	MAFTOOLS	0.1
PICARD_COLLECTMULTIPLEMETRICS	picard	3.1.1
RUN_ICHORCNA	ichorcna	0.3.2
Workflow	Nextflow	24.4.2
	dincalcilab/samurai	v1.0dev-gfd35d1f

dincalcilab/samurai Methods Description

Suggested text and references to use when describing pipeline usage within the methods section of a publication.

Methods

Data was processed using dincalcilab/samurai v1.0dev of the nf-core collection of workflows (Ewels *et al.*, 2020).

The pipeline was executed with Nextflow v24.04.2 (Di Tommaso *et al.*, 2017) with the following command:

```
nextflow run DIncalciLab/swgscna -r master --input samplesheet.csv -params-file parameters.yaml --outdir output_ichorcna/ -profile clus
ter -qs 4
```

References

- Di Tommaso, P., Chatzou, M., Floden, E. W., Barja, P. P., Palumbo, E., & Notredame, C. (2017). Nextflow enables reproducible computational workflows. Nature Biotechnology, 35(4), 316-319. https://doi.org/10.1038/nbt.3820
- Ewels, P. A., Peltzer, A., Fillinger, S., Patel, H., Alneberg, J., Wilm, A., Garcia, M. U., Di Tommaso, P., & Nahnsen, S. (2020). The nf-core framework for community-curated bioinformatics pipelines. Nature Biotechnology, 38(3), 276-278. https://doi.org/10.1038/s41587-020-0439-x

Notes:

- If available, make sure to update the text to include the Zenodo DOI of version of the pipeline used.
- The command above does not include parameters contained in any configs or profiles that may have been used. Ensure the config file is also uploaded with your publication!
- You should also cite all software used within this run. Check the "Software Versions" of this report to get version information.

dincalcilab/samurai Workflow Summary

- this information is collected when the pipeline is started.

Core Nextflow options

revision master
runName pedantic_shirley
containerEngine apptainer
launchDir /home/incalci/lbeltrame/analysis/samurai-ichorcna
workDir /home/incalci/lbeltrame/analysis/samurai-ichorcna/work
projectDir /home/incalci/lbeltrame/.nextflow/assets/DIncalciLab/swgscna
userName lbeltrame
profile cluster
configFiles N/A

Input/output options

input samplesheet.csv
outdir output_ichorcna/

Reference genome options

fasta /opt/bcbio/1.2.8/genomes/Hsapiens/hg38/seq/hg38.fa
fai /opt/bcbio/1.2.8/genomes/Hsapiens/hg38/seq/hg38.fa.fai
dict /opt/bcbio/1.2.8/genomes/Hsapiens/hg38/seq/hg38.dict
igenomes_ignore true

Institutional config options

custom_config_base /home/incalci/shared/pipeline-configs/

Max job request options

max_cpus 100
max_memory 260.GB

Generic options

hook_url https://hookshot.heavensinferno.net/webhook/slack/a154b5f4-5a45-491c-9d4c-

multiqc_logo `/home/incalci/lbeltrame/.nextflow/assets/DIncalciLab/swgscna/assets/dincalcilab-samurai_logo_dark.png`

fffa71e7dd4d

Alignment options

aligner `false`

Common sWGS options

caller `ichorcna`
analysis_type `liquid_biopsy`

Common liquid biopsy options

normal_panel `/home/incalci/shared/references/ctDNA_PoN_median.rds`

ichorCNA options

ichorcna_readcounter_chrs `chr1,chr2,chr3,chr4,chr5,chr6,chr7,chr8,chr9,chr10,chr11,chr12,chr13,chr14,chr15,chr16,chr17,chr18,chr19,chr20,chr21,chr22,chrX`
ichorcna_chrs_to_use `paste0('chr', c(1:22, 'X'))`
ichorcna_chrs_to_train `paste0('chr', c(1:22, 'X'))`
ichorcna_chrs_to_normalize `paste0('chr', c(1:22))`
ichorcna_max_cn `6`
ichorcna_estimate_ploidy `false`
ichorcna_normal_states `0.90, 0.98, 0.99, 0.995, 0.999`
ichorcna_gc_wig `/home/incalci/shared/references/ichorCNA/gc_hg38_500kb.wig`
ichorcna_map_wig `/home/incalci/shared/references/ichorCNA/map_hg38_500kb.wig`
ichorcna_centromere_file `/home/incalci/shared/references/ichorCNA/GRCh38.GCA_000001405.2_centromere_acen.txt`

GISTIC specific options

run_gistic `true`
gistic_t_amp `0.3`

**gistic_t_del**	`0.3`
**gistic_remove_x**	`true`
**gistic_broad_analysis**	`true`
**gistic_broad_chr_length**	`0.7`

MultiQC v1.21 - Written by Phil Ewels, available on GitHub.
This report uses HighCharts, jQuery, jQuery UI, Bootstrap, FileSaver.js and clipboard.js.

seqera
