# Supplementary material for: SAMURAI: shallow analysis of copy number alterations using a reproducible and integrated bioinformatics pipeline
Source: Brief Bioinform. 2025 Jan 29;26(1):bbaf035. doi: 10.1093/bib/bbaf035 (PMC11775468; doi:10.1093/bib/bbaf035)
Supplement: Supplementary_File1_Report_SAMURAI_Dataset_T_Review_bbaf035 [file supplementary_file1_report_samurai_dataset_t_review_bbaf035.pdf]

multiqc v1.21

SAMURAI Results

General Stats
Picard
- Alignment Summary
- Mean read length
- Base Distribution
- Insert Size
- Mean Base Quality by Cycle
- Base Quality Distribution
- WGS Coverage
- WGS Filtered Bases
Gistic arm-level analysis summary
Gistic Analysis
ASCAT.sc Summary
Gistic Analysis Summary
Copy Number Signatures Activities
Software Versions
dincalcilab/samurai Methods Description
dincalcilab/samurai Workflow Summary

multiqc

SAMURAI Results

SAMURAI's analysis results of Copy Number Alterations from shallow whole genome sequencing.

A modular tool to aggregate results from bioinformatics analyses across many samples into a single report.

This report has been generated by the dincalcilab/samurai analysis pipeline.

Report generated on 2024-11-28, 16:47 CET based on data in: `/home/incalci/lbeltrame/analysis/samurai-stage2/work/e2/3e1d51567d1684a41e79e9c3351757`

Welcome! Not sure where to start? Watch a tutorial video (6:06) don't show again

General Statistics

Copy table | Configure columns | Scatter plot | Violin plot | Showing 46/46 rows and 2/6 columns. | Export as CSV

Sample Name	Insert Size	Mean Coverage
1	221 bp	0.2 X
10	242 bp	0.3 X
113_left	105 bp	0.2 X
113_right	105 bp	0.2 X
119	197 bp	0.2 X
12	210 bp	0.3 X
123_left	211 bp	0.4 X
123_right	212 bp	0.4 X
131_left	209 bp	0.2 X
131_right	224 bp	0.3 X
133	221 bp	0.4 X
137	194 bp	0.3 X
148	240 bp	0.4 X
164	234 bp	0.3 X
183_left	100 bp	0.2 X

Picard

Picard is a set of Java command line tools for manipulating high-throughput sequencing data.

Alignment Summary

Please note that Picard's read counts are divided by two for paired-end data. Total bases (including unaligned) is not provided.

Percentages | Aligned Reads | Aligned Bases | Export Plot

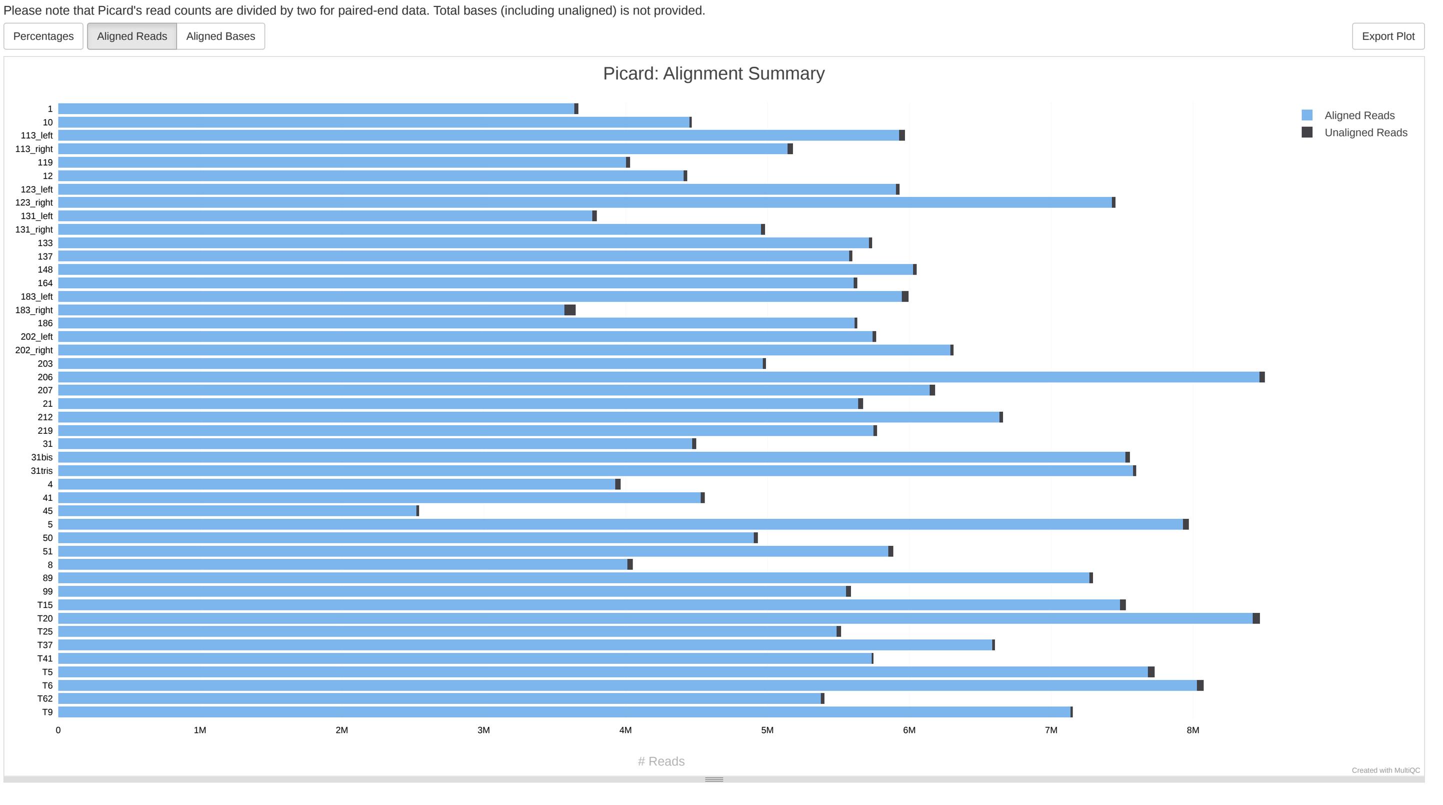

Mean read length

The mean read length of the set of reads examined.

Export Plot

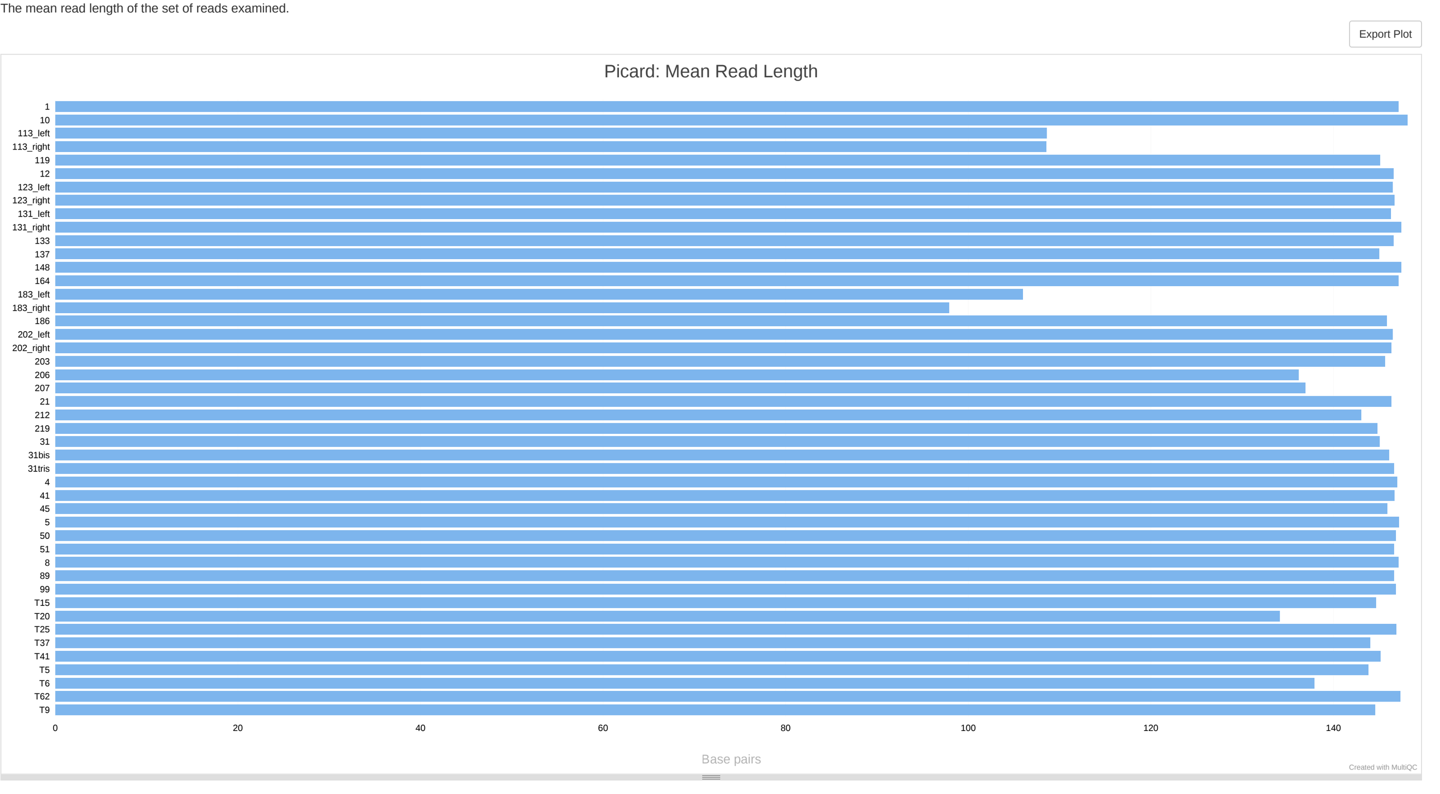

Base Distribution

Plot shows the distribution of bases by cycle.

% Adenine | % Cytosine | % Guanine | % Thymine | % Undetermined | Export Plot

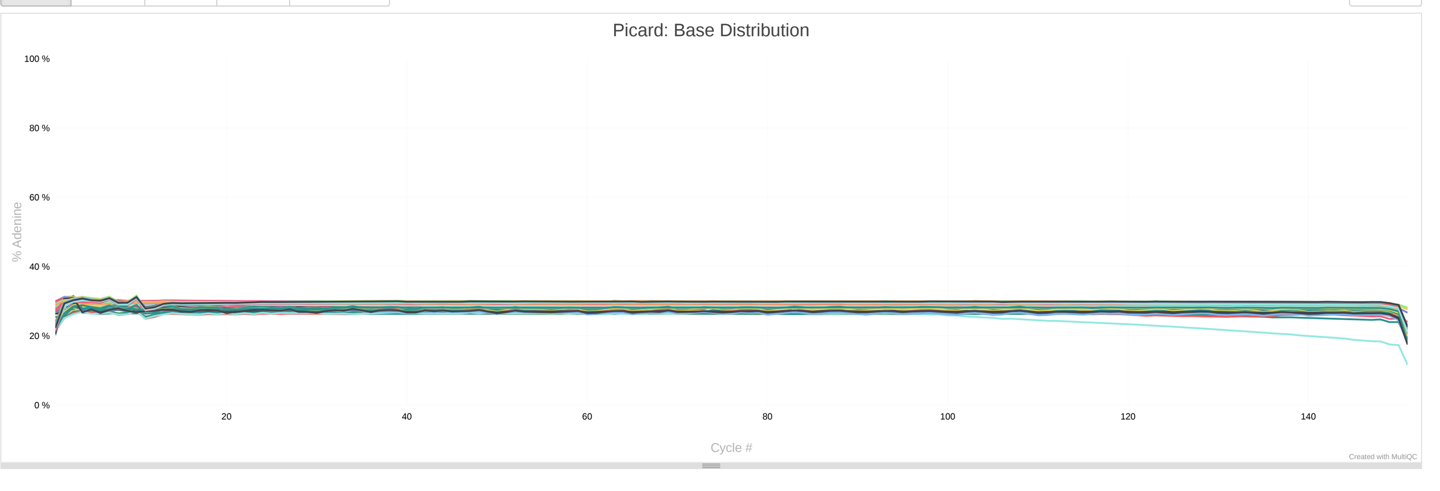

Insert Size

Plot shows the number of reads at a given insert size. Reads with different orientations are summed.

Counts | Percentages | Export Plot

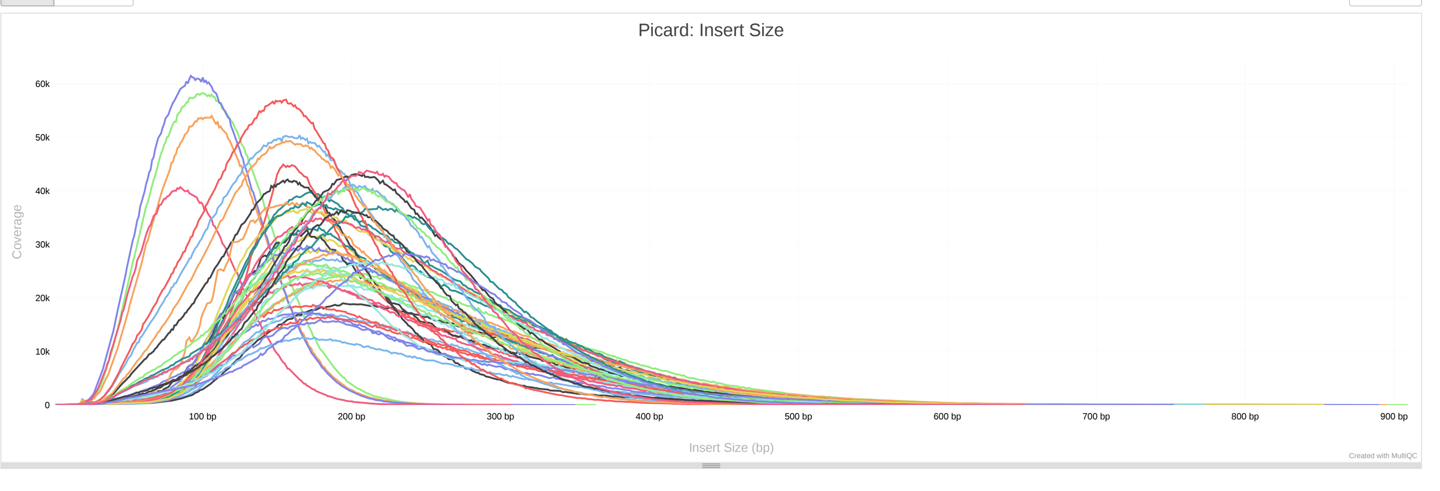

Mean Base Quality by Cycle

Help

Plot shows the mean base quality by cycle.

Export Plot

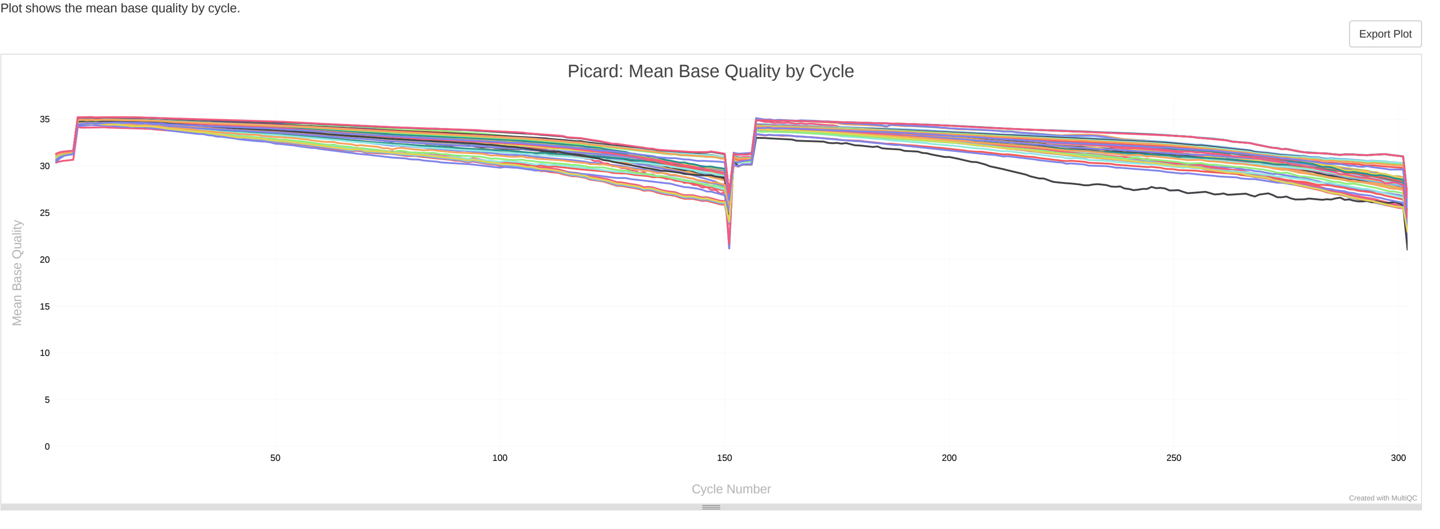

Base Quality Distribution

Plot shows the count of each base quality score.

Export Plot

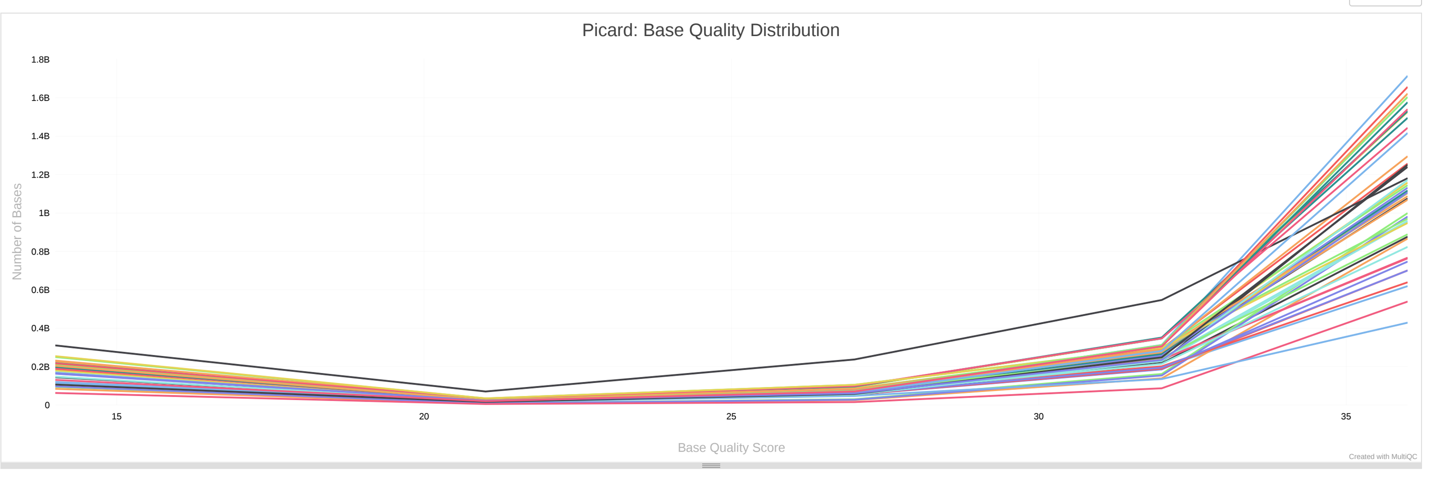

WGS Coverage

The number of bases in the genome territory for each fold coverage. Note that final 1% of data is hidden to prevent very long tails.

Percentage Drop-Off | Counts Histogram | Export Plot

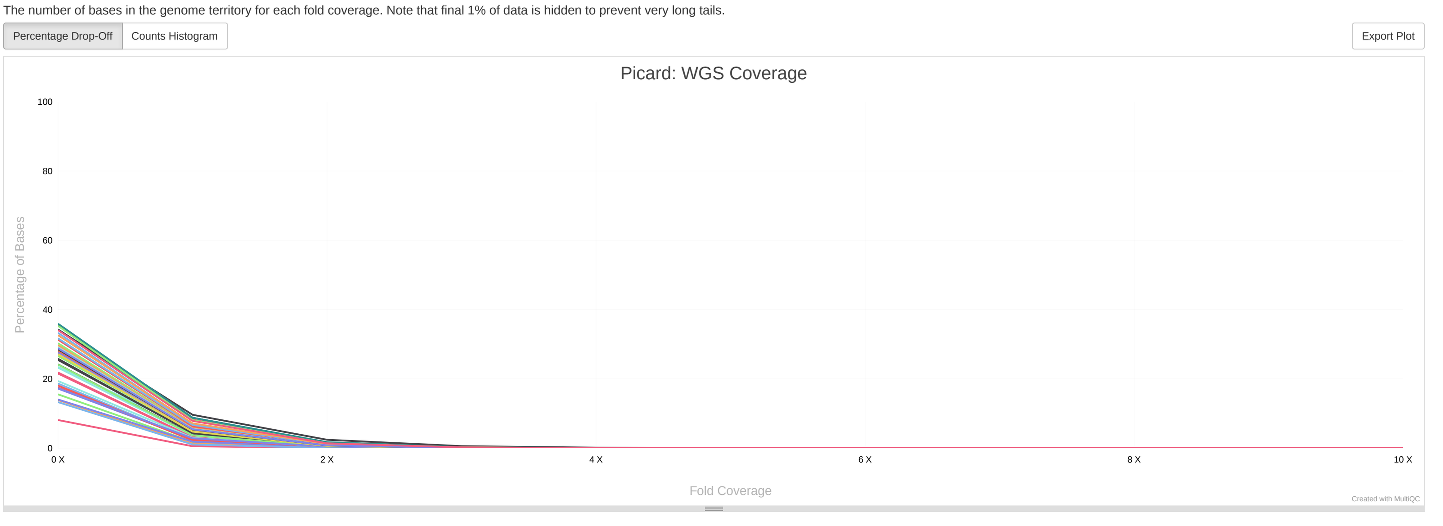

WGS Filtered Bases

For more information about the filtered categories, see the Picard documentation.

Export Plot

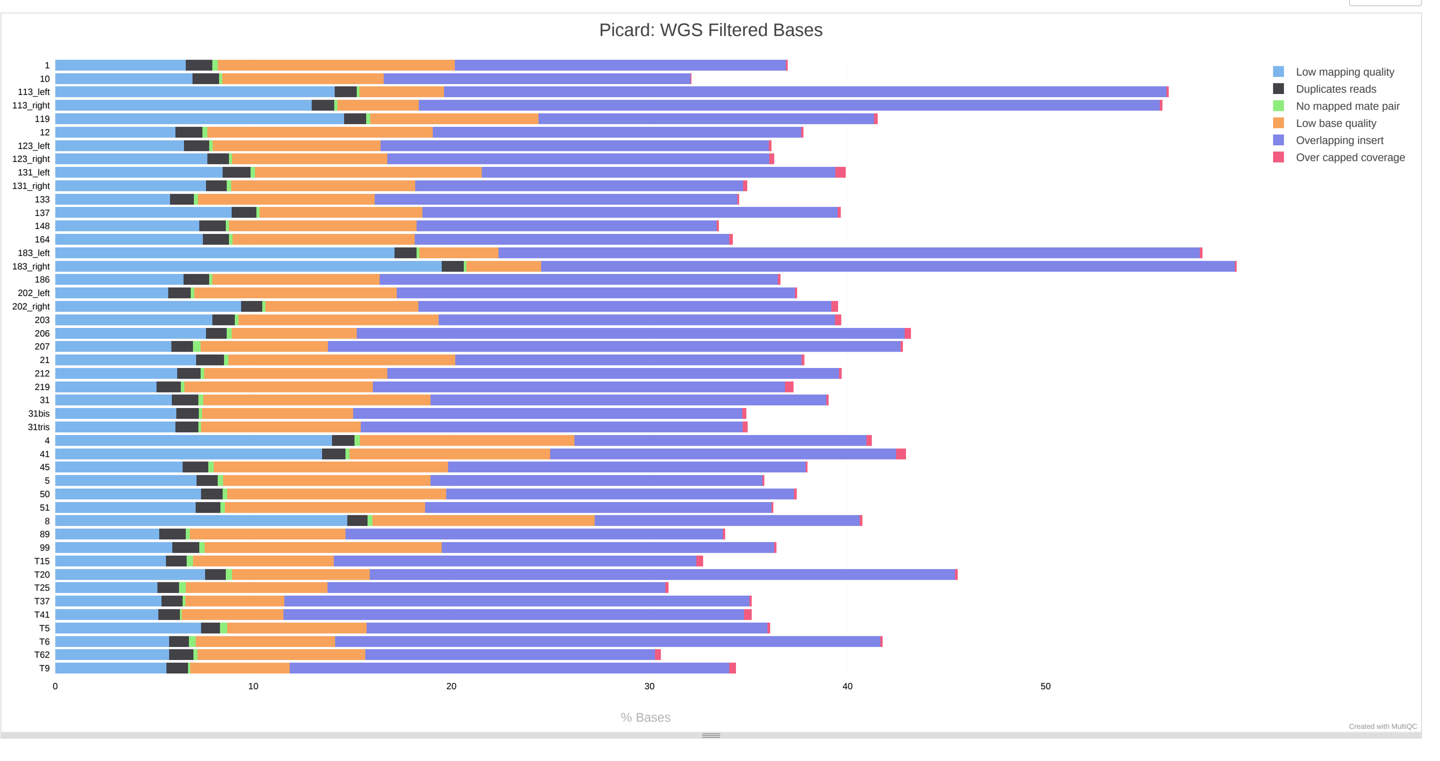

Gistic arm-level analysis summary

This table shows significant recurrent arm-level alterations identified by GISTIC2. Each alteration has an associated frequency and q-value for statistical significance. .

Copy table | Configure columns | Scatter plot | Violin plot | Showing 10/10 rows and 4/4 columns. | Export as CSV

Arm	Type of alteration	No. of samples	Frequency	q-value
4p	Del	20	43.0	0.0145
4q	Del	21	46.0	0.0011
8p	Del	24	52.0	0.0001
13q	Del	22	48.0	0.0012
16p	Del	18	39.0	0.0158
16q	Del	20	43.0	0.0051
17p	Del	33	70.0	0.0000
17q	Del	25	52.0	0.0000
18q	Del	19	41.0	0.0344
22q	Del	21	43.0	0.0097

Gistic Analysis

Maftools genomic plot with segments highlighting significant Amplifications and Deletions.

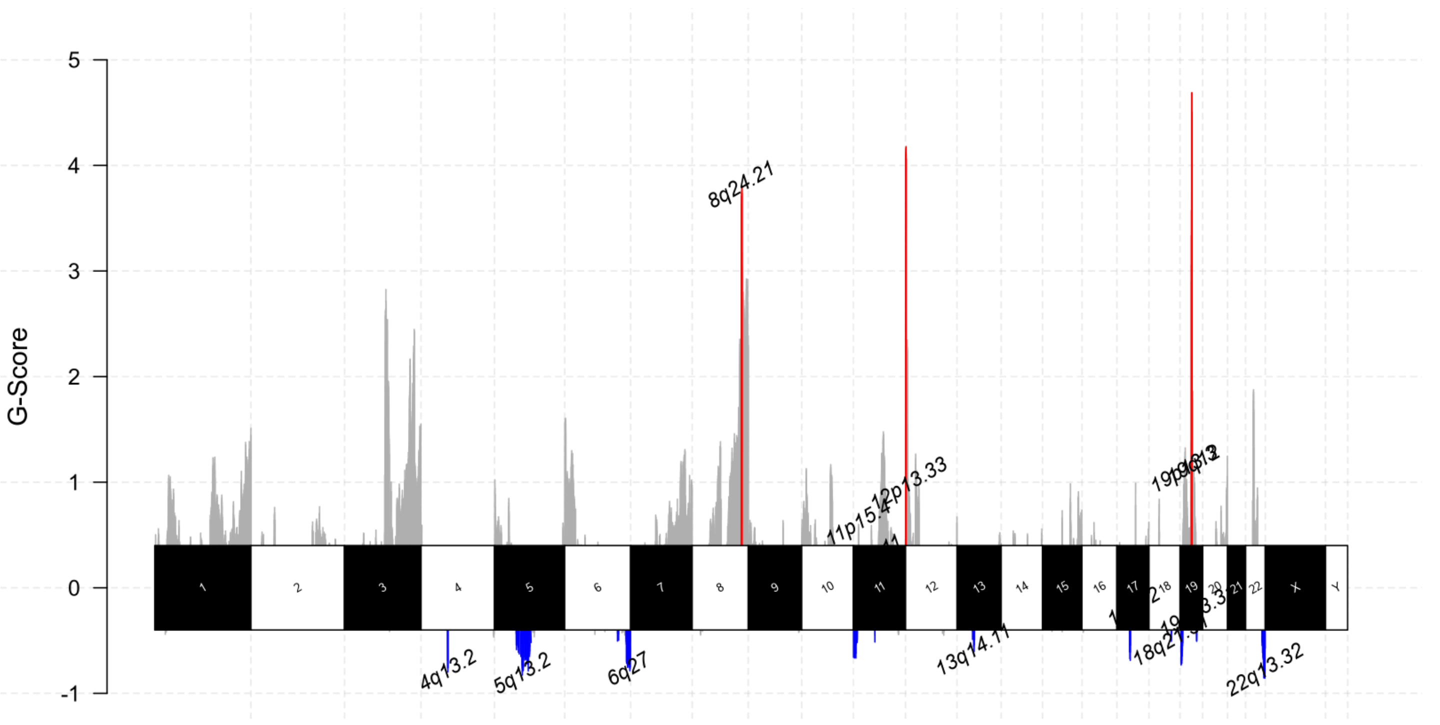

ASCAT.sc Summary

This table shows purity and ploidy values computed by ASCAT.sc.

Copy table | Configure columns | Scatter plot | Violin plot | Showing 46/46 rows and 2/2 columns. | Export as CSV

Sample ID	Purity	Ploidy
T37	0.4700	2.4200
186.0	0.2900	2.7600
T5	1.0000	2.0100
1.0	0.8600	2.8200
99.0	0.9600	3.8600
5.0	0.7800	2.8200
T41	0.7700	1.7900
148.0	0.9200	2.9200
31_Tris	0.9000	4.4900
164.0	0.9000	1.8000
T25	0.7400	3.1400
51.0	0.5000	3.4200
131_right	0.6400	2.7700
50.0	0.7200	3.0900
113_right	0.8300	1.7500

Gistic Analysis Summary

This table shows significant lesions identified by GISTIC2 analysis. Focal lesions are alterations which span less than 25% of a chromosomal arm. Broad lesions span more than 25% of the chromosomal arm, but less than a whole arm .

Copy table | Configure columns | Scatter plot | Violin plot | Showing 14/14 rows and 9/9 columns. | Export as CSV

Chromosome	Chromosome	Cytoband	Start	End	Length	CN Kind	Lesion Frequency	Nr. of Samples	Type of Lesion
Del_4q13.2_chr4:68120002-69420000	chr4	4q13.2	68 120 002	69 420 000	1 299 998	Del	0.50	23	focal
Del_5q13.2_chr5:55290627-94745000	chr5	5q13.2	55 290 627	94 745 000	39 454 373	Del	0.59	27	focal
Del_6q27_chr6:159125002-170805979	chr6	6q27	159 125 002	170 805 979	11 680 977	Del	0.70	32	focal
Amp_8q24.21_chr8:127190558-129010000	chr8	8q24.21	127 190 558	129 010 000	1 819 442	Amp	0.80	37	focal
Del_11p15.4_chr11:1-10565000	chr11	11p15.4	1	10 565 000	10 564 999	Del	0.65	30	focal
Del_11q11_chr11:55370002-55710000	chr11	11q11	55 370 002	55 710 000	339 998	Del	0.33	15	focal
Amp_12p13.33_chr12:760002-1680000	chr12	12p13.33	760 002	1 680 000	919 998	Amp	0.63	29	focal
Del_13q14.11_chr13:31620387-54774926	chr13	13q14.11	31 620 387	54 774 926	23 154 539	Del	0.57	26	focal
Del_17q12_chr17:32765002-34935000	chr17	17q12	32 765 002	34 935 000	2 169 998	Del	0.70	32	focal
Del_18q21.31_chr18:37155063-80373285	chr18	18q21.31	37 155 063	80 373 285	43 218 222	Del	0.61	28	broad
Amp_19q12_chr19:29950002-30550000	chr19	19q12	29 950 002	30 550 000	599 998	Amp	0.43	20	focal
Del_19p13.3_chr19:1-6220000	chr19	19p13.3	1	6 220 000	6 219 999	Del	0.63	29	broad
Del_19q13.31_chr19:42740002-43435000	chr19	19q13.31	42 740 002	43 435 000	694 998	Del	0.48	22	focal
Del_22q13.32_chr22:44505002-50204868	chr22	22q13.32	44 505 002	50 204 868	5 699 866	Del	0.74	34	focal

Copy Number Signatures Activities

The image is a summary view of Copy number Signatures from segmentation files, computed by the R Package CINSignatureQuantification.

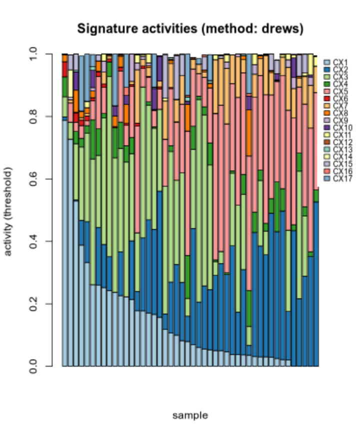

Software Versions

Software Versions lists versions of software tools extracted from file contents.

Copy table

Group	Software	Version
ASCAT_SC	ASCAT.sc	0.1
ASSEMBLE_GISTIC_OUTPUT	gistic-cli	0.4.5
GISTIC2	GISTIC2	0.1
MAFTOOLS	MAFTOOLS	0.1
PICARD_COLLECTMULTIPLEMETRICS	picard	3.1.1
Workflow	Nextflow	24.4.2
	dincalcilab/samurai	v1.0.3-gdf6e344

dincalcilab/samurai Methods Description

Suggested text and references to use when describing pipeline usage within the methods section of a publication.

Methods

Data was processed using dincalcilab/samurai v1.0.3 (doi: 10.1101/2024.09.30.615766) of the nf-core collection of workflows (Ewels *et al.*, 2020).

The pipeline was executed with Nextflow v24.04.2 (Di Tommaso *et al.*, 2017) with the following command:

```
nextflow run DIncalciLab/swgscna -r master --input samplesheet.hgsoc.csv -params-file parameters.yaml --outdir output/ -profile cluster -resume -qs 2
```

References

- Di Tommaso, P., Chatzou, M., Floden, E. W., Barja, P. P., Palumbo, E., & Notredame, C. (2017). Nextflow enables reproducible computational workflows. Nature Biotechnology, 35(4), 316-319. https://doi.org/10.1038/nbt.3820
- Ewels, P. A., Peltzer, A., Fillinger, S., Patel, H., Alneberg, J., Wilm, A., Garcia, M. U., Di Tommaso, P., & Nahnsen, S. (2020). The nf-core framework for community-curated bioinformatics pipelines. Nature Biotechnology, 38(3), 276-278. https://doi.org/10.1038/s41587-020-0439-x

Notes:

- The command above does not include parameters contained in any configs or profiles that may have been used. Ensure the config file is also uploaded with your publication!
- You should also cite all software used within this run. Check the "Software Versions" of this report to get version information.

dincalcilab/samurai Workflow Summary

- this information is collected when the pipeline is started.

Core Nextflow options

revision	master
runName	evil_babbage
containerEngine	apptainer
launchDir	/home/incalci/lbeltrame/analysis/samurai-stage2
workDir	/home/incalci/lbeltrame/analysis/samurai-stage2/work
projectDir	/home/incalci/lbeltrame/.nextflow/assets/DIncalciLab/swgscna
userName	lbeltrame
profile	cluster
configFiles	N/A

Input/output options

input	samplesheet.hgsoc.csv
outdir	output/

Reference genome options

fasta	/opt/bcbio/1.2.8/genomes/Hsapiens/hg38/seq/hg38.fa
fai	/opt/bcbio/1.2.8/genomes/Hsapiens/hg38/seq/hg38.fa.fai
dict	/opt/bcbio/1.2.8/genomes/Hsapiens/hg38/seq/hg38.dict
igenomes_ignore	true

Institutional config options

custom_config_base	/home/incalci/shared/pipeline-configs/

Max job request options

max_cpus	100
max_memory	260.GB

Generic options

hook_url	https://hookshot.heavensinferno.net/webhook/slack/a154b5f4-5a45-491c-9d4c-fffa71e7dd4d
multiqc_logo	/home/incalci/lbeltrame/.nextflow/assets/DIncalciLab/swgscna/assets/dincalcilab-samurai_logo_dark.png

Common sWGS options

binsize	30
caller	ascat_sc

ichorCNA options

ichorcna_chrs_to_use	paste0('chr', c(1:22))
ichorcna_chrs_to_train	paste0('chr', c(1:22))
ichorcna_chrs_to_no…	paste0('chr', c(1:22))

GISTIC specific options

run_gistic	true
gistic_t_amp	0.3
gistic_t_del	0.3
gistic_remove_x	true
gistic_broad_analysis	true
gistic_broad_chr_len…	0.7

Other parameters

compute_signatures	true
